# Fucoidan-Based Theranostic Nanogel for Enhancing Imaging and Photodynamic Therapy of Cancer

**DOI:** 10.1007/s40820-020-0384-8

**Published:** 2020-02-04

**Authors:** Mi Hyeon Cho, Yan Li, Pui-Chi Lo, Hyeri Lee, Yongdoo Choi

**Affiliations:** 1 Division of Translational Science, Research Institute, National Cancer Center, 323 Ilsan-ro, Goyang, Gyeonggi 10408 Republic of Korea; 2 Department of Biomedical Sciences, City University of Hong Kong, Tat Chee Avenue, Kowloon, Hong Kong China

**Keywords:** Fucoidan, Theranostic nanogel, P-selectin, Activatable, Anti-angiogenic

## Abstract

In this study, a fucoidan-based theranostic nanogel (CFN-gel) consisting of a fucoidan backbone, redox-responsive cleavable linker and photosensitizer is developed to achieve activatable near-infrared fluorescence imaging of tumor sites and an enhanced photodynamic therapy (PDT) to induce the complete death of cancer cells. A CFN-gel has nanomolar affinity for P-selectin, which is overexpressed on the surface of tumor neovascular endothelial cells as well as many other cancer cells. Therefore, a CFN-gel can enhance tumor accumulation through P-selectin targeting and the enhanced permeation and retention effect. Moreover, a CFN-gel is non-fluorescent and non-phototoxic upon its systemic administration due to the aggregation-induced self-quenching in its fluorescence and singlet oxygen generation. After internalization into cancer cells and tumor neovascular endothelial cells, its photoactivity is recovered in response to the intracellular redox potential, thereby enabling selective near-infrared fluorescence imaging and an enhanced PDT of tumors. Since a CFN-gel also shows nanomolar affinity for the vascular endothelial growth factor, it also provides a significant anti-tumor effect in the absence of light treatment in vivo. Our study indicates that a fucoidan-based theranostic nanogel is a new theranostic material for imaging and treating cancer with high efficacy and specificity.

**Electronic supplementary material:**

The online version of this article (10.1007/s40820-020-0384-8) contains supplementary material, which is available to authorized users.

## Highlights

- A fucoidan-based theranostic nanogel consisting of a fucoidan backbone, redox-responsive cleavable linker and photosensitizer enables activatable fluorescence imaging of tumor sites and an enhanced photodynamic therapy to induce complete death of cancer.
- To use fucoidan as a polymeric backbone in the nanogel platform enables cancer targeting by P-selectin binding and enhances anti-tumor effect by inhibiting the binding of the vascular endothelial growth factor.

**Electronic supplementary material:**

The online version of this article (10.1007/s40820-020-0384-8) contains supplementary material, which is available to authorized users.

## Graphical abstract

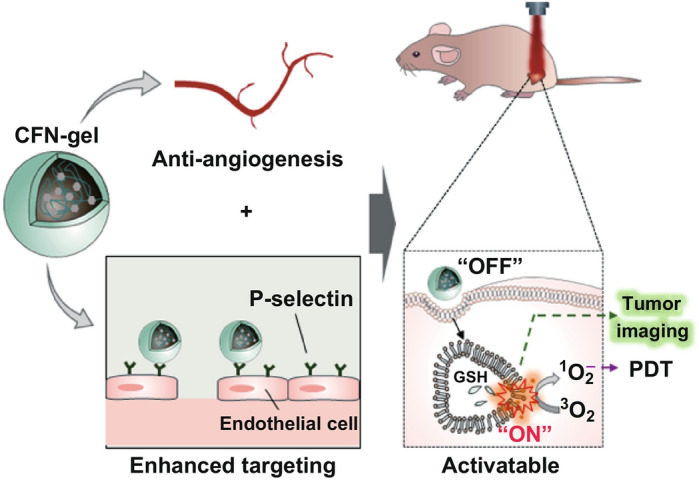

## Introduction

Photodynamic therapy (PDT) has been successfully applied to the treatment of various malignant diseases including cancer, which uses light in combination with oxygen and a light-sensitive drug (i.e., photosensitizer) to destroy cancer cells [1]. When photosensitizers are exposed to a specific wavelength of light, they produce a reactive oxygen species (ROS), including singlet oxygen (^1^O_2_), and rapidly kill the nearby cancer cells [2]. Despite its excellent therapeutic effect, the application of PDT has been limited by the unfavorable biodistribution due to the hydrophobic nature of the photosensitizer (PS), a poor tumor specificity and an unwanted phototoxic effect in normal tissues [3–5]. To overcome these limitations, PS-combined nanoparticles have been demonstrated; these nanoparticles can improve the water solubility and prolong the in vivo circulation time of the PS, thereby enhancing the tumor accumulation compared with free PS molecules [6–8]. For example, nanoparticles (or nanogels) prepared from PS–hyaluronic acid conjugates have shown enhanced PDT due to the increased tumor accumulation of nanoparticles via enhanced permeation and retention (EPR) effect and CD44 receptor targeting. Nevertheless, polymeric materials, which have been used as a backbone for PS conjugation and nanoparticle preparation, have no anti-tumor effects, and therefore, no additional benefits could be obtained with the previously reported polymeric nanosystems in PDT. Therefore, despite the substantial advances of new strategies and methodologies in the field of cancer treatment, challenges regarding their efficacy and specificity still remain.

**Fig. 1:**
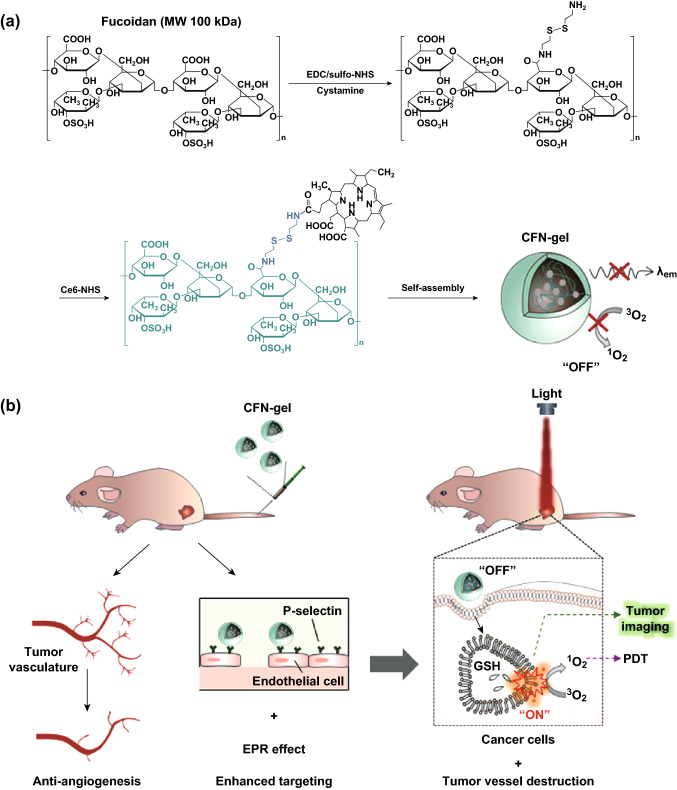
**a** Synthetic steps of Ce6–fucoidan theranostic nanogel (CFN-gel), **b** schematic illustration of CFN-gel and its mode of action

Herein, we describe the development of a fucoidan-based theranostic nanogel (CFN-gel), which comprises a biocompatible fucoidan backbone and chlorin e6 (Ce6) molecules, to achieve activatable fluorescence imaging of tumor sites and an enhanced PDT to induce complete death of cancer cells. Fucoidan is a natural biopolymer with a highly negative charge on a sulfated polysaccharide backbone. In recent years, fucoidan has attracted considerable interest due to its anticancer potential through various mechanisms such as the modulation of a scavenger receptor, metastasis blockade and immune system activation [9–11]. Fucoidan is also known to target the P-selectin, which is overexpressed on the surface of tumor neovascular endothelial cells as well as many other cancer cells [12–14]. In addition to the targeting of P-selectin, the nanoscale dimensions of a CFN-gel are beneficial for the enhancement of tumor accumulation due to the prolonged systemic circulation in the bloodstream and the EPR effect [15]. Therefore, it is expected that fucoidan can enhance the efficacy of the imaging and PDT by providing significant benefits, not only as a hydrophilic polymer backbone to form biocompatible nanogels but also as the targeting ligand of tumor vasculature and cancer cells. Herein, Ce6 is attached to the fucoidan through a disulfide linker, which releases Ce6 in response to the difference in redox potential between the blood stream and cancer cells (Fig. 1a). Owing to the aggregation of hydrophobic Ce6 molecules, Ce6–fucoidan conjugates form self-assembled nanogels in an aqueous solution, and thereafter, the near-infrared (NIR) fluorescence emission and singlet oxygen generation (SOG) of the conjugated Ce6 can be suppressed (OFF) through an aggregation-induced self-quenching effect during blood circulation in normal tissues. When the CFN-gel penetrates the tumor tissue, it enters the cancer cells and tumor neovascular endothelial cells. Then its photoactivity is expected to be recovered (ON) due to the release of Ce6 from the CFN-gel in response to the intracellular redox potential, thereby achieving a selective NIR fluorescence imaging and an activatable PDT of tumors (Fig. 1b). In addition, fucoidan has been found to inhibit the binding of the vascular endothelial growth factor (VEGF), which is a key angiogenesis promoting molecule, to its cell membrane receptor [16–18]. Therefore, we expect that a CFN-gel may provide a significant anti-tumor effect without light illumination, and the residual tumors after PDT as well as the tumors that are located at unknown sites can be treated by a CFN-gel in systemic circulation (Fig. 1b). Overall, a fucoidan-based theranostic nanogel may have the potential utility as a new theranostic material for selective imaging and enhanced cancer therapy with high efficacy and specificity.

## Experimental Section

### Materials

The Ce6 applied was purchased from Frontier Scientific, Inc. (Logan, UT, USA). Fucoidan purified from the sporophyll of *Undaria pinnatifida* was kindly provided by Haerim Fucoidan Co., Ltd (Wando, Republic of Korea). According to the manufacturer’s information, the average molecular weight of the fucoidan was 150 kDa and contained 21 ± 3% fucose, 20 ± 5% galactose, 2 ± 2% mannose, 30 ± 3% sulfate and 25.46% uronic acid. Dithiothreitol (DTT), cystamine dihydrochloride, ethyl-(dimethyl aminopropyl) carbodiimide (EDC), dimethyl sulfoxide (DMSO) and dimethylformamide (DMF) were purchased from Sigma-Aldrich (St. Louis, USA). The singlet oxygen sensor green (SOSG) reagent and sulfo-*N*-hydroxysuccinimide (sulfo-NHS) were obtained from ThermoFisher Scientific (IL, USA). Dialysis membranes (MW cutoff, 15 kDa) were purchased from Spectrum Laboratories (CA, USA). In addition, CCK-8 cell viability assay kits were obtained from Dojindo (Kumamoto, Japan). Recombinant human VEGF 165 and recombinant human P-selectin/CD62P Fc chimera were purchased from PeproTech (Rocky Hill, NJ, USA) and R&D Systems (Minneapolis, MN, USA), respectively.

### Preparation of CFN-Gel

To prepare the Ce6–fucoidan conjugates, amine-functionalized fucoidan was synthesized by conjugating cystamine dihydrochloride with the carboxylic acid groups of fucoidan using EDC/NHS chemistry. Briefly, fucoidan (3.02 µmol) was dissolved in 18 mL of a sodium phosphate buffer (PBS; 10 mM, pH 7.4, 137 mM NaCl). Then, 120 µmol of EDC and 125 µmol of sulfo-NHS were added to the fucoidan solution and reacted at 25 °C. After 30 min, cystamine dihydrochloride (120 µmol), dissolved in 1 mL of a PBS, was added to the activated fucoidan solution. The reaction was carried out for 18 h at 25 °C. After the reaction, the solution was dialyzed against deionized (DI) water for purification and then freeze-dried. For the conjugation of Ce6 into the amine-functionalized fucoidan, the carboxylic acid group of Ce6 (33.5 µmol) was activated using EDC (340 µmol) and sulfo-NHS (350 µmol) in a DMSO solvent. Then, the activated Ce6 solution was mixed with amine-functionalized fucoidan (0.754 µmol) in a DMF:H_2_O co-solvent (5 mL, 1:1 v/v) and then reacted for 18 h at 25 °C. The resulting Ce6–fucoidan solution was dialyzed against a phosphate buffer (pH 7.4, 10 mM) and DI water for 1 day, and then freeze-dried. The final product was analyzed using UV/Vis spectrometry (DU730, Beckman Coulter, Brea, USA), a multi-mode microplate reader (Tecan, Safire2, Switzerland) and proton nuclear magnetic resonance (^1^H-NMR; JEOL JNM-LA400 with LFG, JEOL, Japan).

### Characterization of CFN-Gel

The morphology of the CFN-gel was observed through transmission electron microscopy (TEM; JEM-F200, JEOL Ltd, Japan) after staining with phosphotungstic acid (2%, w/v). The hydrodynamic size and surface charge of the CFN-gel were measured using a zeta potential/particle sizer (Malvern Instrument, Malvern, UK). For the analyses of the optical properties, the CFN-gel was dissolved in PBS (pH 7.4) and surfactant-containing DI water (0.1 M NaOH/0.1% SDS). Fluorescence spectra of the solutions (*λ*_ex_ = 400 nm) were obtained on a multi-mode microplate reader (Tecan, Safire2). For the measurement of the Ce6 concentration, the absorbance of the CFN-gel dissolved in surfactant-containing DI water (0.1 M NaOH/0.1% SDS) was measured at 400 nm (with a molar extinction coefficient of Ce6 = 1.5 × 10^5^ M^−1^ cm^−1^ at 400 nm) [19].

### Redox-Responsive Turn-on of NIR Fluorescence and SOG

To evaluate the redox-responsive fluorescence turn-on of the CFN-gel upon treatment of a reducing agent, a CFN-gel in PBS at a concentration of 2 µM Ce6 equivalent was prepared. The time-dependent changes in the fluorescence spectrum of the CFN-gel solution (*λ*_ex_ = 400 nm; *λ*_em_ = 600–800 nm) were then measured periodically after the addition of 0, 5 µM and 5 mM DTT. SOSG was used to detect the redox-responsive turn-on of SOG by the CFN-gel. The PBS solution used in this test was pre-saturated with an oxygen gas. The CFN-gel was dissolved in PBS and then treated with DTT for 4 h. Concentrated SOSG was added to the DTT-treated sample solutions. The final concentration of the SOSG reagent in the test solutions was adjusted to 5 µM. Finally, the solutions were irradiated using a 670-nm CW laser (50 mW cm^−2^); meanwhile, the increase in the SOSG fluorescence in the samples was measured every 30 s. All experiments were conducted in quadruplicate.

### Surface Plasmon Resonance (SPR) Analysis

An SPR (Biacore T200) analysis was applied for the measurement of the binding affinity of the CFN-gel to VEGF 165 and P-selectin, individually. Immobilization of recombinant human VEGF 165 and recombinant human P-selectin/CD62P Fc chimera was conducted on an individual CM5 sensor chip using an amide coupling method. The resulting immobilization units for the VEGF 165 and P-selectin were 2800 RU and 791 RU, respectively. Samples were diluted in HBS-EP running buffer. The sensorgrams were analyzed using BIAevaluation software.

### Cell Culture

The HT1080 human fibrosarcoma cell line and primary HDF were obtained from the American Type Culture Collection (Rockville, MD, USA). These cell lines were cultured in Eagle’s minimum essential media and Dulbecco’s modified Eagle’s medium, respectively, which contains 10 (v/v)% FBS and 1% antibiotic–anti-mycotic.

### Confocal Fluorescence Microscopy for Intracellular Uptake

HT1080 cells were seeded at a density of 5 × 10^4^ cells well^−1^ onto a Lab-Tek II Chambered Coverglass and incubated for 24 h for cell attachment. Then, free Ce6 and a CFN-gel were diluted in a cell culture medium containing 10% FBS to obtain the equivalent concentrations of 2 µM Ce6. After incubation for 6 h, the cells were washed three times and a fresh culture medium was added. NIR fluorescence images of the Ce6 and CFN-gel (*λ*_ex_ = 633 nm, *λ*_em_ = 650 nm, using a long-pass filter) were acquired using confocal scanning laser microscopy (CSLM, ZEISS LSM 780). For a quantification of the intracellular uptake, the acquired images were analyzed using ZEN software (ZEISS, Jena, Germany) to convert the image into numerical values.

### In Vitro Dark Toxicity Test

HT1080 cancer cells were seeded in 96-well plates at a density of 1 × 10^4^ cells well^−1^ and incubated for 24 h to allow cell attachment. The Ce6 and CFN-gel were diluted in a cell culture medium containing 10% FBS to concentrations of 0.1, 1, 2.5, 5, 10 and 20 µM. The cells were treated with a culture medium containing various concentrations of Ce6 and CFN-gel for 6 h. The cells were then washed three times, and a fresh cell culture medium without photosensitizers was added. After further incubating the cells for 24 h, the cell viability (*n* = 4 per group) was analyzed using a CCK-8 assay kit.

For comparison, an in vitro dark toxicity test of the CFN-gel was conducted in HDF as a normal cell line. HDF was seeded in 96-well plates at a density of 1 × 10^4^ cells well^−1^ and incubated for 24 h. The Ce6 and CFN-gel were diluted in a cell culture medium containing 10% FBS to concentrations of 0.1, 1, 2.5, 5, 7.5, 10 and 20 µM. The HDF cells were treated with the culture medium containing various concentrations of Ce6 and CFN-gel for either 6 or 24 h. Then, the cells were washed three times, and a fresh cell culture medium was added. After further incubating the cells for 24 h, the cell viability was analyzed.

### In Vitro Phototoxicity Testing

HT1080 cells were plated on 96-well plates at density of 1 × 10^4^ cells well^−1^ and incubated for 24 h. The cells were treated with a culture medium containing various concentrations of Ce6 and a CFN-gel (0.01, 0.1, 1, 2.5, 5, 7.5 and 10 µM) for 6 h. All cells were then washed three times, and a fresh culture medium was added. The cells were irradiated with a 670-nm CW laser at 10 J cm^−2^ (irradiation dose rate of 50 mW cm^−2^) for PDT. After further incubating the cells for 24 h, the cell viability was analyzed.

### In Vivo Studies in a Xenograft Tumor Model

Male BALB/c nude mice (6 weeks olds) were used for the in vivo experiments. All animal experiments were approved by the Institutional Animal Care and Use Committee. HT1080 cells (5 × 10^6^ cells 0.1 mL^−1^ of Hank’s balanced salt solution) were subcutaneously implanted into the right flank of each mouse, and the size of the tumor was measured daily.

For an in vivo NIR fluorescence imaging study, six mice with HT1080 tumors of ~ 200 mm^3^ in size received intravenous injections of the free Ce6 or CFN-gel (5 mg Ce6 equiv. kg^−1^ 100 μL^−1^ PBS; *n* = 3 per group). For comparison, the mice in the control group (*n* = 3) received intravenous injections of 100 μL PBS. Then, NIR fluorescence images (*λ*_ex_ = 660/20 nm, *λ*_em_ = 710/40 nm) were obtained using the IVIS Lumina imaging system at 5 min and 24 h after injection. For biodistribution analysis, the mice were killed at 24 h post-injection and ex vivo NIR fluorescence imaging of the tumors and organs (spleen, kidneys, the liver, lung and heart) were carried out. After that, the collected tumor tissues were immediately frozen and cryo-sectioned at a 7 μm thickness. After staining the cell nuclei in the tumor sections with DAPI (Vector Laboratories, Inc., Burlingame, USA), fluorescence images of tumor tissues were obtained using a confocal scanning laser microscope (Carl Zeiss LSM 780; *λ*_ex_ = 405 nm and *λ*_em_ = 420–480 nm for DAPI; *λ*_ex_ = 633 nm and *λ*_em_ = 647–754 nm for Ce6). Immunohistochemical staining of CD62P in the tumor sections was also conducted using an anti-CD62P (R&D systems, Minneapolis, USA) primary antibody to demonstrate the P-selectin expression.

For the in vivo tumor growth study, 24 mice with HT1080 tumors of ~ 70 mm^3^ in size were divided into four groups on day zero: group 1, PBS (100 μL, *n* = 7); group 2, free Ce6 (5 mg Ce6 kg^−1^) + PDT (*n* = 5); group 3, CFN-gel (5 mg Ce6 equiv. kg^−1^) alone (*n* = 7); and group 4, CFN-gel (5 mg Ce6 equiv. kg^−1^) + PDT (*n* = 5). PBS, free Ce6 or a CFN-gel was injected intravenously on day 1. After 24 h (i.e., day 2), the tumor sites in groups 2 and 4 were irradiated using a 670-nm laser for PDT (50 mW cm^−2^, 20 J cm^−2^). On day 10, all mice were killed and their organs (i.e., heart, lung, liver, spleen and kidney) were collected. The tissues were then sectioned and stained with H&E for a histopathological analysis.

To further analyze the anti-tumor effect by PDT, another set of 12 tumor-bearing mice were used for a histopathological analysis of apoptotic cell death in the tumors. After treating the mice as mentioned above, tumor tissues (*n* = 3 per group) were harvested on day 3 and sectioned. A terminal deoxynucleotidyl transferase dUTP nick end labeling (TUNEL) assay was performed using a TdT-FragEL DNA fragmentation detection kit (Promega, Madison, USA).

Next, the anti-angiogenic effect by the CFN-gel itself was evaluated from an immunohistochemical analysis of CD31 staining of the tumor sections. Six mice with tumors received an intravenous injection of either PBS (three mice, 100 μL) or a CFN-gel (three mice, 5 mg Ce6 equiv. kg^−1^) on day 1. Tumor tissues were collected and sectioned on day 7. The tumor sections were stained with an anti-CD31 (Abcam, Cambridge, USA) primary antibody to visualize the tumor blood vessels. The changes in angiogenic blood vessels in the tumors were quantitatively analyzed by counting the number of CD31-positive endothelial cells in the tumor sections. The mean (S.E.) of the stained blood vessel endothelial cells was calculated from five different digital images. For apoptosis analysis, TUNEL assay of the tumor sections was performed. The mean (S.E.) of the stained cancer cells was calculated from six different digital images.

### Serum Biochemical Analysis

Athymic nude mice without tumors received an intravenous injection of PBS (100 μL, *n* = 4), free Ce6 (5 mg Ce6 kg^−1^, *n* = 4) and CFN-gel (5 mg Ce6 equiv. kg^−1^, *n* = 4), and serum samples were then collected on day 10 to analyze the levels of alanine aspartate aminotransferase (AST), aminotransferase (ALT), alkaline phosphatase (ALP), glucose (Glu), total bilirubin (T-Bili), blood urea nitrogen (BUN), creatinine (Crea), total cholesterol (T-Chol), triglycerides (TG), total protein (TP), albumin (Alb) and the A/G ratio.

### Statistical Analysis

All data are expressed as mean ± S.D. except for the CD31 and TUNEL analyses. A Student’s *t* test was carried out to analyze the significant difference between test groups.

## Results and Discussion

### Characterization of CFN-Gel

**Fig. 2:**
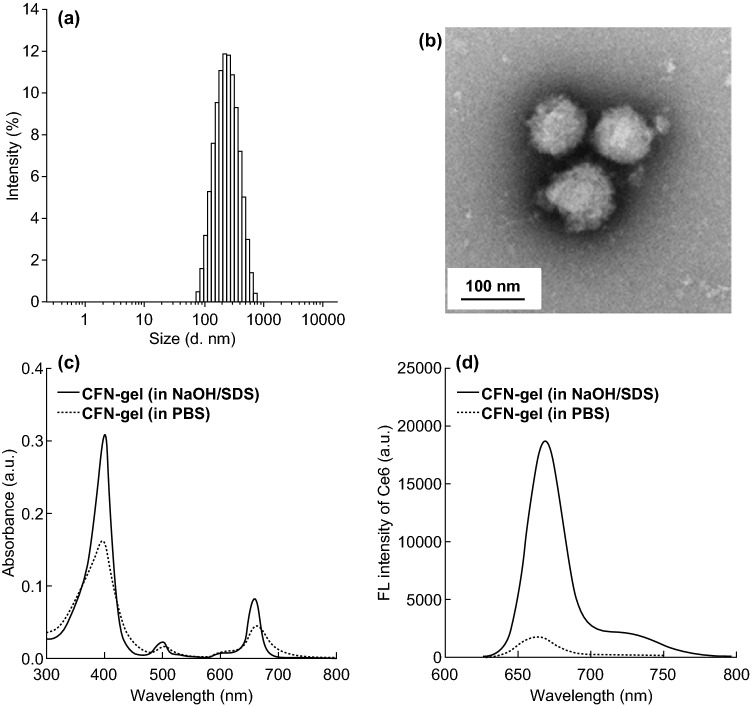
Characterization of CFN-gel. **a** Hydrodynamic size distribution of CFN-gel, **b** TEM image of CFN-gel, **c** UV/Vis absorption spectra of CFN-gels in surfactant-containing (0.1 M NaOH/1% SDS) (solid line) and PBS (dotted line) solutions at a 2 µM Ce6 equivalent, **d** fluorescence spectra (*λ*_ex_ 400 nm) of CFN-gel in surfactant-containing (solid line) and PBS (dotted line) solutions at 2 µM Ce6 equivalent

To prepare the Ce6–fucoidan nanogel (CFN-gel), carboxylic acids of the fucoidan backbone were first modified with cystamine dihydrochloride using EDC/sulfo-NHS chemistry, as shown in Fig. 1a. The Ce6 was then conjugated into an amino-terminated fucoidan. According to the ^1^H-NMR analysis of the Ce6–fucoidan conjugate, the number of Ce6 photosensitizers per fucoidan backbone was calculated to be 20.3 (Fig. S1). When the Ce6–fucoidan conjugate was freeze-dried and re-dispersed in an aqueous solution, its hydrodynamic size and zeta potential in an aqueous solution were found to be 259 $\pm$ 59 nm and − 22.2 mV, respectively (Fig. 2a). This indicates that the Ce6–fucoidan conjugate forms self-assembled nanogels (i.e., CFN-gel) in an aqueous solution owing to the hydrophobic interaction and subsequent aggregation between the conjugated Ce6 molecules. The morphological analysis of a CFN-gel observed through TEM also confirmed the formation of the nanogels (Fig. 2b). The UV/Vis absorption spectrum of a CFN-gel in a phosphate-buffered saline (PBS; 10 mM, pH 7.4, 137 mM NaCl) solution showed a broadening of the Soret band region, which is a hallmark of an aggregated photosensitizer [20]. After the disintegration of a CFN-gel by dissolving it in a surfactant-containing solution (0.1 M NaOH/1% SDS solution), its absorption spectrum recovers to that of the Ce6 molecules in a free from without an aggregation (Fig. 2c). It is well known that the aggregation of PSs causes the effective quenching of both the fluorescence emission and SOG [20]. Indeed, the fluorescence emission of a CFN-gel in a PBS solution was quenched (i.e., turn-off) by tenfold compared to a CFN-gel in a surfactant-containing solution (Fig. 2d).

### Redox-Responsive Turn-on of NIR Fluorescence and SOG

**Fig. 3:**
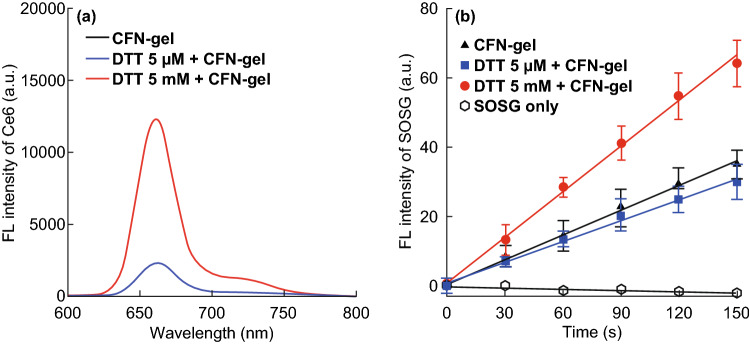
Redox-responsive turn-on of fluorescence signal and singlet oxygen generation of CFN-gel. **a** Fluorescence spectra of CFN-gel treated with DTT (0, 5 µM or 5 mM) for 4 h (*λ*_ex_ = 400 nm), **b** time-dependent increase in SOSG fluorescence of DTT-treated CFN-gel during 670-nm laser irradiation (*n* = 4)

It is expected that the quenched status of a CFN-gel can be restored upon degradation of the nanogels by responding to the concentration gradient of the reducing agents between the extracellular and intracellular environments. That is, the redox-responsive cleavage of the disulfide linkers in the CFN-gel inside the cells can trigger the release and disaggregation of Ce6 molecules from the nanogel, thereby inducing a turn-on fluorescence emission and SOG (Figs. 3a and S2). Therefore, the CFN-gel was incubated under various concentrations of the reducing agent, dithiothreitol (DTT, 0–5 mM), at 37 °C for 4 h, and the change in fluorescence intensity of the CFN-gel was monitored. As shown in Figs. S2 and 3a, there were no significant changes in the fluorescence of the CFN-gel in the absence of DTT or in the presence of 5 μM DTT. However, when the CFN-gel was treated with 5 mM DTT, its fluorescence intensity was increased by 4.9-fold, implying that the aggregated Ce6 molecules in the CFN-gel can be effectively released upon cleavage of the disulfide linkers, and hence, the CFN-gel fluorescence could be turned on. Next, we evaluated whether the SOG capability of the CFN-gel can be recovered by responding to the change in the redox potential (Fig. 3b). To analyze the SOG from a CFN-gel during light irradiation, singlet oxygen sensor green (SOSG) was used as a singlet-oxygen-detecting reagent. The CFN-gel was first treated with different concentrations of DTT for 4 h and then mixed with SOSG, followed by a measurement of the change in fluorescence intensity of SOSG upon 670-nm laser irradiation. As shown in Fig. 3b, the treatment of 5 mM DTT increased the SOG of a CFN-gel by 1.9-fold compared with that of a CFN-gel treated with 0 or 5 μM DTT. These data indicate that a CFN-gel can exhibit a redox-responsive fluorescence emission and phototoxicity.

### SPR Analysis of CFN-Gel

**Fig. 4:**
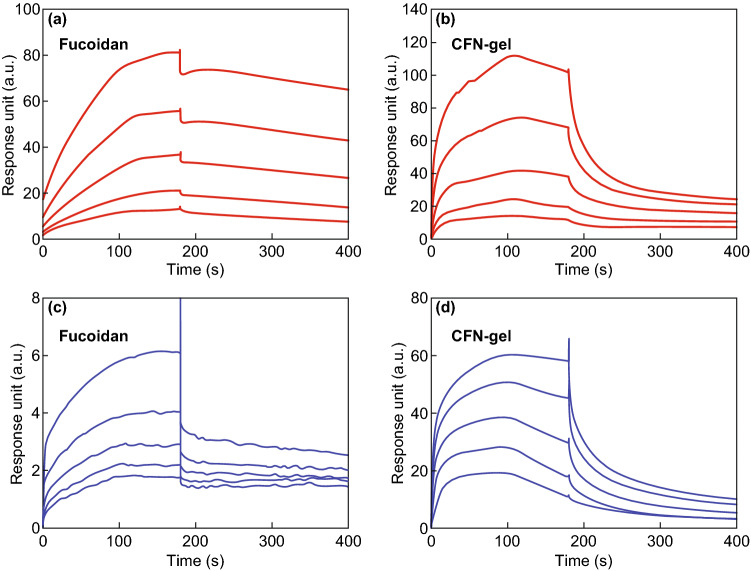
Surface plasmon resonance (SPR) analysis. **a** Sensorgram of SPR for evaluating the binding affinity between fucoidan and VEGF 165 (equilibrium dissociation rate constant (*K*_D_) = 953.0 nM) and CFN-gel and VEGF 165 (*K*_D_ = 756.1 nM), **b** sensorgram of SPR for evaluating the binding affinity between fucoidan and P-selectin (*K*_D_ = 69.71 nM), and between CFN-gel and P-selectin (*K*_D_ = 718.9 nM)

The binding characteristics of a CFN-gel to VEGF 165 and P-selectin were compared using a SPR analysis. Different concentrations of 5, 2.5, 1.25, 0.625 and 0.3125 µM of fucoidan and a CFN-gel were analyzed, and the sensorgrams are presented in Fig. 4. The equilibrium dissociation rate constant (*K*_D_) of the fucoidan and CFN-gel for VEGF 165 was calculated as 953.0 and 756.1 nM, respectively (Fig. 4a). The *K*_D_ of the fucoidan and CFN-gel for P-selectin was calculated as 69.71 and 718.9 nM, respectively (Fig. 4b). These results confirmed that the CFN-gel has a nanomolar affinity for both VEGF 165 and P-selectin.

### In Vitro Cell Studies

Prior to in vitro cell studies, the dispersion stability of CFN-gel was tested in cell culture medium containing 10% fetal bovine serum (Fig. S3). No apparent aggregation or precipitation of CFN-gel was observed for 240 h of incubation, indicating the good dispersion stability of the nanogels in the cell culture medium containing serum proteins.

**Fig. 5:**
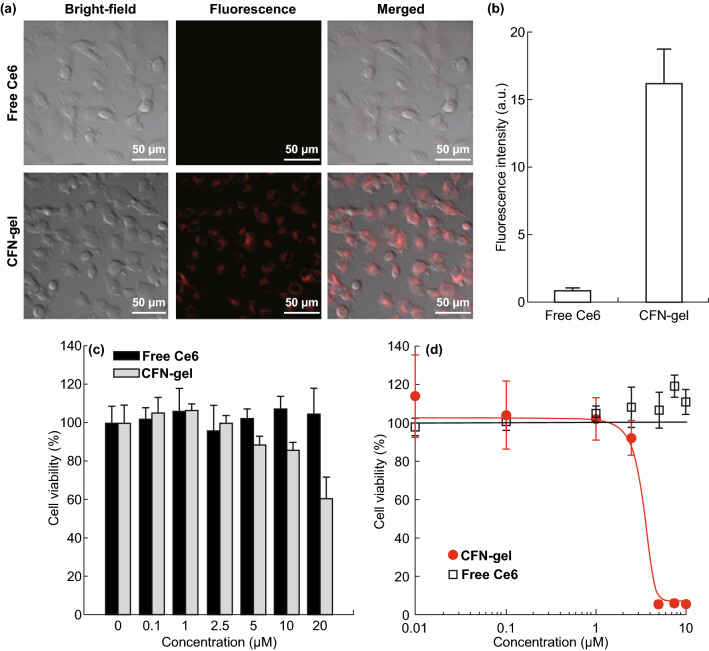
**a** NIR fluorescence images of free Ce6 and CFN-gel-treated HT1080 cancer cells. The HT1080 cancer cells were treated with free Ce6 or CFN-gel at a 2 µM Ce6 equivalent for 6 h. After washing the cells, NIR fluorescence images (*λ*_ex_ = 633 nm, *λ*_em_ = 650, using a long-pass filter) were obtained. The red color indicates the fluorescence signals generated from Ce6, **b** quantitative analysis of fluorescence intensities in the free Ce6 and CFN-gel-treated HT1080 cells (*n* = 4), **c** in vitro dark cytotoxicity of the HT1080 cells after treatment with free Ce6 and CFN-gel at various concentrations, **d** in vitro phototoxicity of the HT1080 cells after treatment with free Ce6 and CFN-gel at various concentrations upon 670-nm light irradiation (50 mW cm^−2^, 10 J cm^−2^). The IC_50_ of the CFN-gel is 2.73 µM. (Color figure online)

To observe the intracellular uptake of a CFN-gel in cancer cells, HT1080 cancer cells were treated with free Ce6 and a CFN-gel at a 2 μM Ce6 equivalent for 6 h. In the confocal fluorescence microscopic images (Fig. 5a), a strong NIR fluorescence was observed in the CFN-gel-treated cells, whereas a minor fluorescence was detected in the free Ce6-treated cells, implying that the CFN-gel was efficiently internalized into the cancer cells, and the quenched Ce6 fluorescence was turned on after the redox-responsive release of Ce6 inside the cells. A quantitative analysis of Ce6 fluorescence in the confocal images indicates that there was at least an 18-fold higher intracellular uptake of CFN-gels than free Ce6 (Fig. 5b). The in vitro therapeutic efficacy of a CFN-gel in HT1080 cancer cells was explored by comparing the cell viability percentages using a CCK-8 assay (Fig. 5c). In the absence of light exposure, there was no cytotoxicity in the cancer cells treated with free Ce6 for 6 h under the tested concentrations. Similarly, no cytotoxicity was observed for the CFN-gel-treated cancer cells at concentrations of 0.1 to 2.5 μM. However, as the concentration of the CFN-gel increased from 5, 10 and 20 μM, the cell viability gradually decreased to 88.3%, 85.9% and 60.2%, respectively. In addition, the treatment of a CFN-gel for 24 h further reduced the cell viability, whereas the treatment of the cancer cells with free Ce6 for 24 h did not show any cytotoxic effect in the cancer cells at up to 10 μM (Fig. S4). When applying a CFN-gel to the human primary dermal fibroblast cells (HDF) as a model of normal cells for either 6 or 24 h, no cytotoxicity was observed (Fig. S5). Such dose-dependent cytotoxicity of a CFN-gel against cancer cells may be attributed to the anticancer activity of the fucoidan itself, as previously reported [9, 10]. Next, the PDT efficacy of a CFN-gel was examined (Fig. 5d). HT1080 cells were incubated using a CFN-gel or free Ce6 for 6 h under various concentrations and irradiated with a 670-nm laser at a light dose of 10 J cm^−2^ (dose rate, 50 mW cm^−2^). The cells were then incubated for another 24 h. Notably, the concentration of conjugated Ce6 in a CFN-gel for 50% death of the cancer cells (IC_50_) under light irradiation was approximately 2.73 μM in the HT1080 cells. In contrast, free Ce6-treated cells showed no therapeutic effect upon light irradiation.

### In Vivo NIR Fluorescence Imaging

**Fig. 6:**
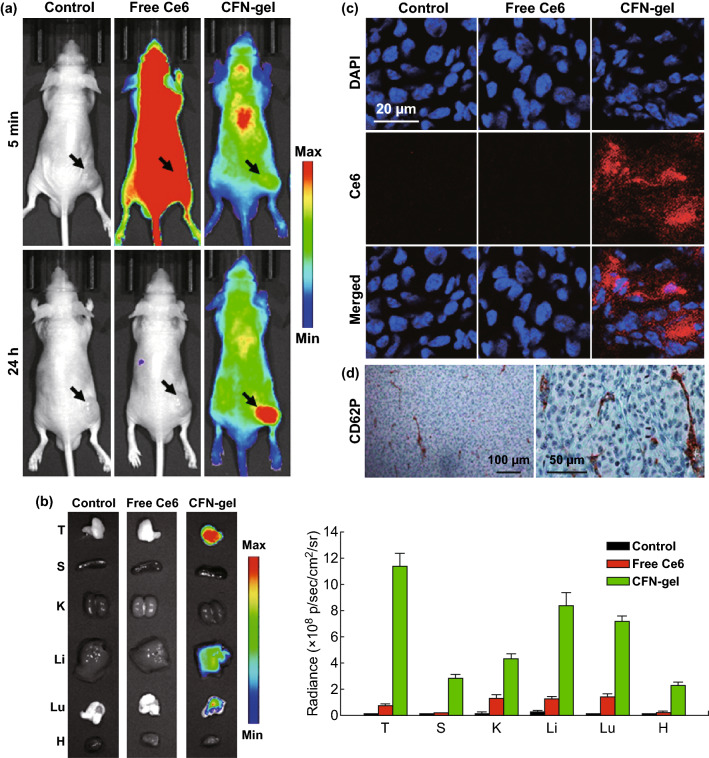
In vivo evaluation of selective tumor imaging in xenograft tumor models. **a** NIR fluorescence images of PBS-, free Ce6- or CFN-gel-treated mice. After 5 min and 24 h of intravenous injection of PBS (100 μL), free Ce6 (5 mg Ce6 kg^−1^) or a CFN-gel (5 mg Ce6 equiv. kg^−1^), NIR fluorescence images of the HT1080 tumor-bearing mice were obtained (*λ*_ex_ = 660/20 nm, *λ*_em_ = 710/40 nm), **b** left: ex vivo NIR fluorescence images of the tumors and major organs (T: tumor, S: spleen, K: kidney, Li: liver, Lu: lung, H: heart) at 24 post-injection. Right: quantitative analysis comparison of fluorescent intensity in the tissues (*n* = 3 per group), **c** confocal fluorescence microscopic images of tumor sections prepared 24 h post-injection. Nuclei in the tumor sections were stained using DAPI. The fluorescence of DAPI (*λ*_ex_ = 405 nm, *λ*_em_ = 420–480 nm) and Ce6 (*λ*_ex_ = 633 nm, *λ*_em_ = 647–754 nm) were pseudocolored in blue and red, respectively, **d** immunohistochemical staining images of CD62P expression (i.e., P-selectin) in the tumor sections. (Color figure online)

To assess the utility of a CFN-gel for in vivo cancer imaging, we used a xenograft mouse tumor model. The HT1080 cells were subcutaneously implanted into the right flank of each mouse, and the tumors were allowed to grow to approximately 200 mm^3^. Following intravenous injection with PBS (three mice, 100 µL mouse^−1^), free Ce6 (three mice, 5 mg kg^−1^) and CFN-gel (three mice, 5 mg Ce6 equiv. kg^−1^), NIR fluorescence images (*λ*_ex_ = 660/20 nm, *λ*_em_ = 710/40 nm) were obtained at 5 min and 24 h after injection (Fig. 6a). At 5 min post-injection, free Ce6-treated mice showed high fluorescence intensities over the entire body, whereas low fluorescence signals were observed in the CFN-gel-treated mice. At 24 h post-injection, minor fluorescence signals were observed in the free Ce6-treated mice, implying that most of the Ce6 was cleared from the body without a significant accumulation of free Ce6 in the tumor tissues. In contrast, fluorescence signals were still observed over the entire body in CFN-gel-treated mice at 24 h post-injection, and the tumor site was clearly discriminated from the surrounding normal tissues. This indicates that the injected CFN-gel showed not only prolonged blood circulation and subsequent accumulation in the tumor tissues but also fluorescence turn-on, which was consistent with the in vitro studies. The tumor tissues and major organs were collected 24 h post-injection to further study the accumulation of the CFN-gel and fluorescence turn-on in the tumor tissues. As shown in Fig. 6b, c, a strong Ce6 fluorescence was observed in the tumors and tumor sections of the CFN-gel-treated mice, whereas no appreciable fluorescence signals of Ce6 were detected in the PBS- and free Ce6-treated mice. As mentioned above, it is known that a high P-selectin expression has been found in the endothelial cells of a tumor vasculature as well as cancer cells in numerous human tumors, but not in adjacent normal tissues [15]. According to the CD62P staining of HT1080 tumor sections, a high P-selectin expression has also been observed (Fig. 6d). It is expected that this P-selectin overexpression in tumor tissues may enhance the tumor-targeting property of a CFN-gel in addition to the EPR effect.

### Anti-tumor Effect of CFN-Gel

**Fig. 7:**
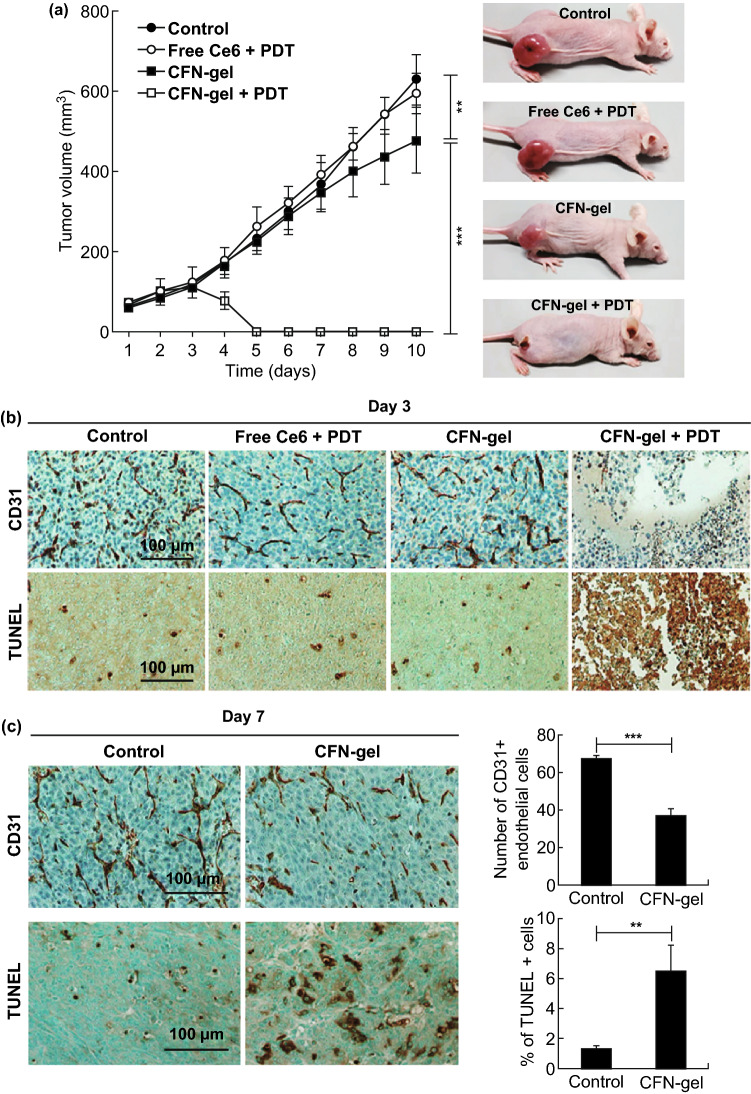
Anti-tumor activity of CFN-gel combined with PDT in vivo. **a** Left: tumor growth curves of mice. Control group (*n* = 7), free Ce6 + PDT (*n* = 5), CFN-gel (*n* = 7) and CFN-gel + PDT (*n* = 5). PBS, free Ce6 or Ce6–fucoidan was injected intravenously on day 1. Light illumination (670 nm, 50 mW cm^−2^, 20 J cm^−2^) of the tumors was conducted on day 2 for PDT. Right: representative photographs of the mice obtained on day 10, **b** immunohistochemical staining of CD31 (for tumor blood vessels) and TUNEL (for apoptotic cell death) of tumor sections collected from different groups of mice at 24 h after PDT, **c** left: micrographs of immunohistochemical staining of CD31 and TUNEL-stained tumor sections collected from control and CFN-gel groups of mice at day 7. Right: quantitative analysis of CD31 and TUNEL assays in the tumor sections. **P < 0.01, ***P < 0.001

The anticancer efficacy of a CFN-gel combined with PDT was further evaluated by measuring the tumor growth rates in a xenograft tumor model of HT1080. When the tumor sizes of the mice reached ~ 70 mm^3^ (day 0), 24 mice were divided into four groups as follows: group 1, PBS (100 μL, *n* = 7); group 2, free Ce6 (5 mg kg^−1^) + PDT (*n* = 5); group 3, CFN-gel (5 mg Ce6 equiv. kg^−1^) alone (*n* = 7); and group 4, CFN-gel (5 mg Ce6 equiv. kg^−1^) + PDT (*n* = 5). PBS, free Ce6 or a CFN-gel was injected intravenously on day 1. After 24 h (i.e., day 2), tumor sites in groups 2 and 4 were irradiated with a 670-nm laser for PDT (50 mW cm^−2^, 20 J cm^−2^). Thereafter, the tumor volumes were measured daily. As a result, remarkably enhanced in vivo therapeutic effects were observed in the CFN-gel + PDT group as compared with the PBS, free Ce6 + PDT and CFN-gel-treated groups (Fig. 7a). On day 10, the mean tumor sizes in groups 3 and 4 were 76.0% (**P < 0.01) and 0% (***P < 0.001), respectively, as compared with those of group 1 (i.e., PBS-treated control group). No apparent tumor mass was detected for any of the five mice in the CFN-gel + PDT-treated group from days 5 to 10. A similar therapeutic trend of the PDT was observed in the CD31 and TUNEL staining of the tumor sections on day 3 (Fig. 7b). According to the TUNEL assays, a dramatic therapeutic effect by a CFN-gel and light irradiation was confirmed, whereas no apparent apoptotic signals were detected in the tumor sections of the other groups (Fig. 7b). Interestingly, angiogenic tumor blood vessels were barely observed from the CD31 staining of the tumor sections of group 4. Based on the data in Figs. 4b and 6d, this is likely due to the CFN-gel being internalized into the P-selectin overexpressing endothelial cells of the tumor vessels and destroying them during light irradiation.

We noticed that the mice treated with a CFN-gel alone (i.e., group 3) showed delayed tumor growth compared with the PBS-treated control group on days 7–10 (Fig. 7a), which might be caused by the anticancer potential of the fucoidan itself [9, 10]. Because the mean tumor sizes in this group were significantly reduced to 76.0% (*P* < 0.01) compared with that of group 1 (i.e., the PBS-treated control group) on day 10, a CD31 staining assay for blood vessels was conducted on day 7 to evaluate the anti-angiogenic effect by the CFN-gel in the tumors (Fig. 7c). Another set of tumor-bearing mice received an intravenous injection of either PBS (100 μL mouse^−1^) or a CFN-gel (5 mg of Ce6 equiv. kg^−1^). The tumor tissues were then collected on day 7, sectioned and stained with anti-CD31 antibodies to visualize the angiogenic blood vessels in the tumors. As shown in Fig. 7c, a quantitative analysis of the immunohistochemical staining images showed a 45.3% decrease in the number of CD31-positive endothelial cells in the CFN-gel-treated tumors as compared with that in the PBS-treated tumors (***P <0.001), supporting an anti-angiogenic effect of the CFN-gel. In addition, the tumor sections were also stained with the TUNEL assay kit for apoptosis analysis. As a result, a 4.9-fold increase in apoptotic cells was observed in the CFN-gel-treated tumors compared to the PBS-treated tumors (***P <0.01), indicating that the CFN-gel accumulated in the tumors induced an anticancer effect, as observed in Figs. 5c and S4a.

### Biocompatibility of CFN-Gel in Vivo

**Fig. 8:**
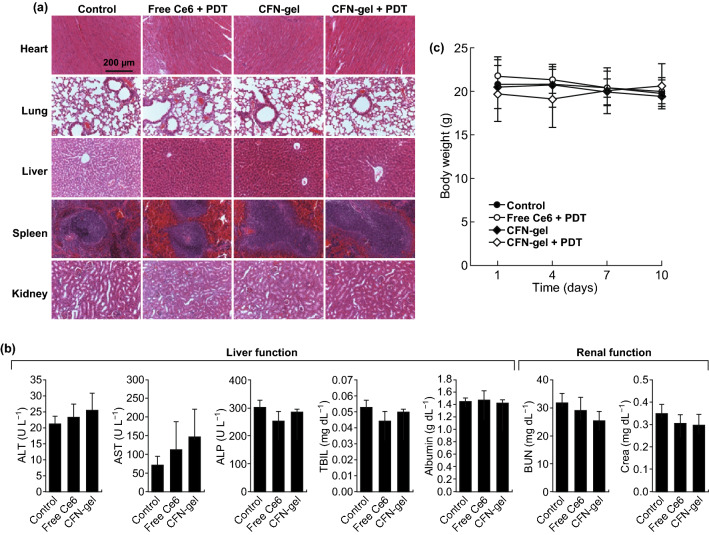
In vivo biocompatibility of CFN-gel. **a** Representative H&E-stained images of major organs collected from different treatment groups on day 10, **b** blood chemistry data of mice in the PBS-, free Ce6- and CFN-gel-treated groups on day 10 (*n* = 4), **c** change in average body weight of mice in different groups over time. No apparent signs of toxicity in organs, blood biomarkers and body weights were observed in any treatment group

We then checked whether the CFN-gel has a potential in vivo toxicity to normal cells or organs (Fig. 8). According to the hematoxylin and eosin (H&E) staining of the tissue sections, no histopathological abnormalities were observed in the major organs (i.e., the heart, lung, liver, spleen or kidney) of all treated groups on day 10 (Fig. 8a). Furthermore, when the blood chemistry profiles of the free Ce6 or CFN-gel-treated groups were analyzed on day 10 and compared with those of the PBS-treated control group (Fig. 8b; Table S1), the levels of the tested blood biomarkers, including those of the liver and kidney toxicities, were not significantly changed by the treatment with the free Ce6 or CFN-gel [21], indicating the in vivo biocompatibility of the CFN-gel. The body weights of the mice in each group did not show a statistical difference during the experimental period (Fig. 8c).

## Conclusions

We developed a fucoidan-based theranostic nanogel (CFN-gel) for selective tumor imaging and enhanced PDT. A CFN-gel itself can provide an anti-angiogenic effect and an enhancement of the tumor accumulation through P-selectin targeting and the EPR effect. A CFN-gel is non-fluorescent and non-phototoxic upon systemic administration. After cellular internalization, its photoactivity can be recovered in response to the intracellular redox potential, thereby enabling selective NIR fluorescence tumor imaging and an enhanced PDT of cancer cells both in vitro and in vivo. Our study indicates that a fucoidan-based theranostic nanogel is a new theranostic material for imaging and treating cancer with high efficacy and specificity. In addition, encapsulation of anticancer drugs inside the CFN-gel can further enhance the cancer therapy by enabling fluorescence image-guided photodynamic/chemo dual therapy [22].

## Electronic supplementary material

Below is the link to the electronic supplementary material.

Supplementary material 1 (PDF 404 kb)
