# Supplementary material for: Fucoidan-Based Theranostic Nanogel for Enhancing Imaging and Photodynamic Therapy of Cancer
Source: Nanomicro Lett. 2020 Feb 4;12:47. doi: 10.1007/s40820-020-0384-8 (PMC7770685; doi:10.1007/s40820-020-0384-8)
Supplement: Supplementary file 1 — Supplementary material 1 (PDF 404 kb) [file 40820_2020_384_MOESM1_ESM.pdf]

Supporting Information for

Fucoidan-based Theranostic Nanogel for Enhancing Imaging and Photodynamic Therapy of Cancer

Mi Hyeon Cho[1, #], Yan Li[1, #], Pui-Chi Lo[2], Hyeri Lee[1], Yongdoo Choi[1, *]

[1]Division of Translational Science, Research Institute, National Cancer Center, 323 Ilsan-ro, Goyang, Gyeonggi 10408, Republic of Korea

[2]Department of Biomedical Sciences, City University of Hong Kong, Hong Kong, SAR China

[#]Mi Hyeon Cho and Yan Li contributed equally to this work

*Corresponding author. E-mail: ydchoi@ncc.re.kr (Yongdoo Choi)

Supplememtary Figures

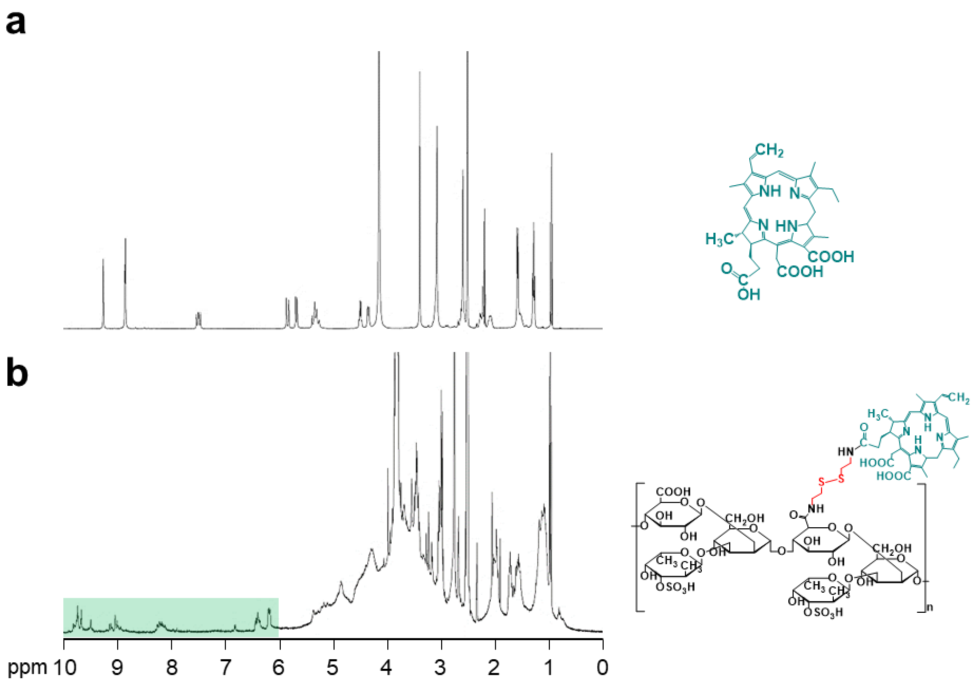

Fig. S1 ^{1}H-NMR spectra of (**a**) Ce6 and (**b**) CFN-gel dissolved in d_6-DMSO/D_2O. The degree of conjugation of Ce6 per a fucoidan molecule was calculated by comparing the peak area at the two intense unresolved peaks at 0.96 and 1.12 ppm (corresponding to the methyl groups of fucose units) and 6.0–7.0 ppm (corresponding to -CH=CH2 of Ce6) of ^{1}H-NMR spectrum [S1-S3]

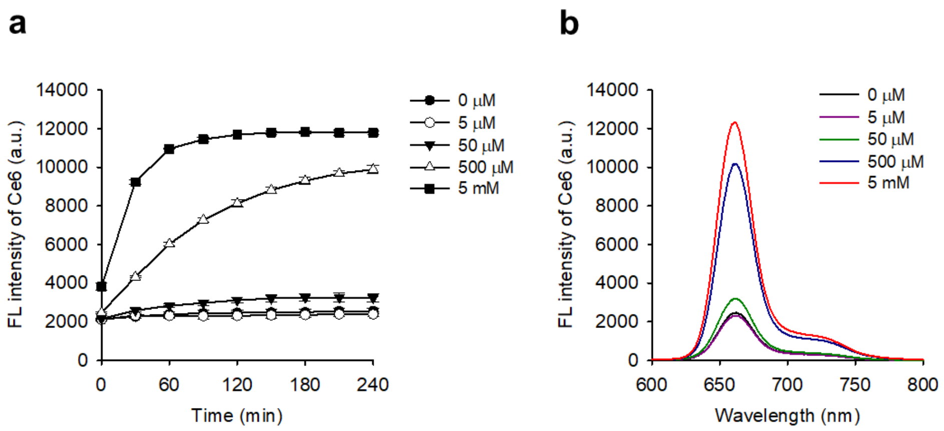

Fig. S2 (**a**) Recovery in NIR fluorescence of CFN-gel over time after addition of various concentrations of reducing agent DTT. (**b**) Fluorescence spectra of CFN-gel at 2 µM of Ce6 equivalent after incubation in 0-5 mM DTT solution for 4 h (λ_{ex} = 400 nm)

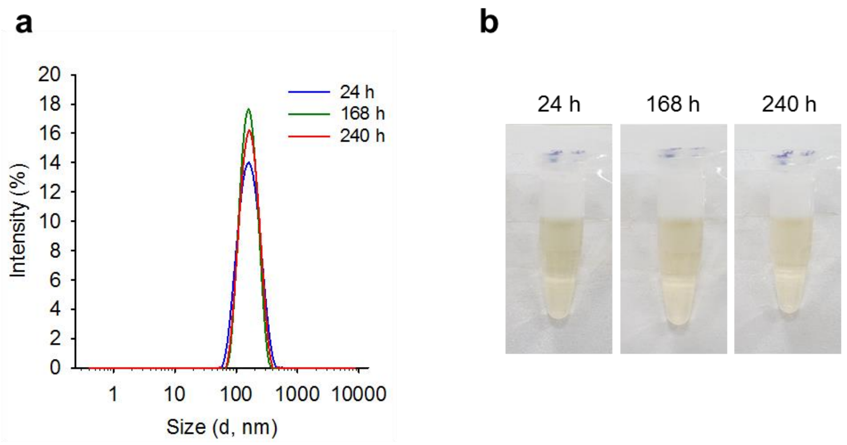

Fig. S3 Dispersion stability of CFN-gel in the DMEM cell culture medium (without phenol red) containing 10% FBS. (**a**) Hydrodynamic sizes and size distributions of CFN-gel which were measured at 24, 168, and 240 h. (**b**) Photographs of sample solutions acquired at 24, 168, and 240 h post-incubation. No precipitate of CFN-gel was observed during 240 h of incubation time, indicating good dispersion stability of the nanogels

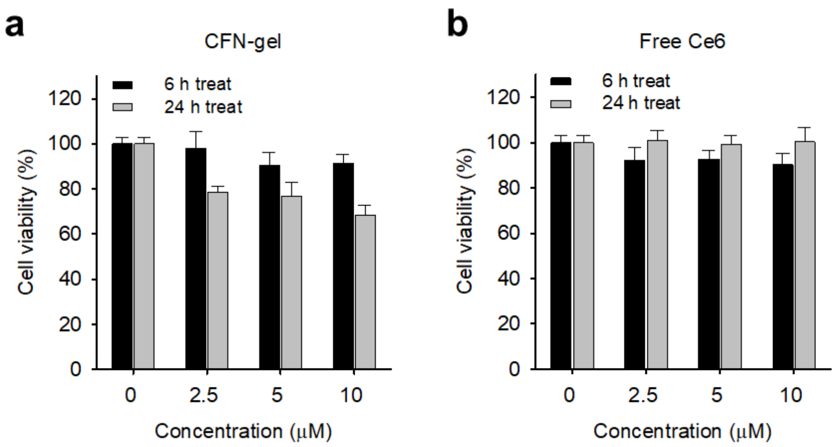

Fig. S4 In vitro cell viability of HT1080 cells after treated with CFN-gel and free Ce6 at various concentrations without light irradiation

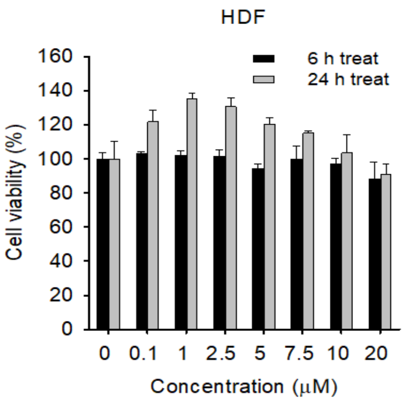

Fig. S5 Viability of normal cells such as human primary dermal fibroblast cells (HDF) after treated with CFN-gel at various Ce6 equivalent concentrations. No significant change in cell viability was observed at all the tested concentrations of CFN-gel compared with that of untreated control HDF

Table S1 Effects of CFN-gel on blood chemistry in mice

	Control (n=4)	Free Ce6 (n=4)	CFN-gel (n=4)
ALT (U/L)	21.15±2.34	23.13±4.37	25.43±5.35
AST (U/L)	71.05±24.04	112.65±74.72	146.93±74.66
ALP (U/L)	300.48±27.21	252.13±34.55	284.03±9.62
Glu (mg/dL)	263.25±47.55	238.75±16.40	275.25±52.98
BUN (mg/dL)	31.83±3.36	28.98±4.67	25.33±3.38
Crea (mg/dL)	0.35±0.04	0.31±0.04	0.30±0.05
T-Bili (mg/dL)	0.015±0.010	0.023±0.026	0.040±0.014
T-chol (mg/dL)	60.50±7.94	56.25±10.87	63.25±9.71
TG (mg/dL)	125.00±19.88	76.75±12.87	86.00±2.71
TP (g/dL)	4.28±0.17	4.35±0.44	4.13±0.17
Alb (g/dL)	1.45±0.06	1.487±0.15	1.43±0.05
A/G ratio	0.52±0.02	0.52±0.05	0.53±0.05

Abbreviations: ALT, alanine aminotransferase; AST, aspartate aminotransferase; ALP, alkaline phosphatase; Glu, glucose; BUN, blood urea nitrogen; Crea, creatinine; T-Bili, total bilirubin; T-Chol, total cholesterol; TG, triglycerides; TP, total protein; Alb, albumin.

Supplementary References

[S1]L. Chevolot et al., Further data on the structure of brown seaweed fucans: relationships with anticoagulant activity. Carbohydr. Res. **319**, 154–165 (1999). http://dx.doi.org/10.1016/S0008-6215(99)00127-5

[S2]B. Mulloy et al., Sulfated fucans from echinoderms have a regular tetrasaccharide repeating unit defined by specific patterns of sulfation at the 0-2 and 0-4 positions. J. Biol. Chem. **269**, 22113–22123 (1994).

[S3]S. Jung et al., Hyaluronic Acid-Conjugated with Hyperbranched Chlorin e6 Using Disulfide Linkage and Its Nanophotosensitizer for Enhanced Photodynamic Therapy Cancer Cells. Mater. **12**, 3080-3102 (2019). http://dx.doi.org/10.3390/ma12193080
